# Supplementary material for: Development of a computer-aided design software for the quantitative evaluation of aesthetic damage
Source: PLoS One. 2019 Dec 18;14(12):e0226322. doi: 10.1371/journal.pone.0226322 (PMC6919621; doi:10.1371/journal.pone.0226322)
Supplement: S1 Code site — 1. (ZIP) [file pone.0226322.s001.zip › [EESC_jr][Projeto]Relatorio_07_21/reports/30/2017.05.16/13:08:53/report.pdf]

Preâmbulo

Na data de 16/05/2017 no horário , o(a) Perito(a) Me. Geraldo Elias Miranda, cirurgião-dentista, designado(a)para proceder ao exame pericial solicitado em: Jose , do sexo , nascido(a) em 01/01/1970, ocupação , portador(a) do RG , cujo endereço , passa a expor a avaliação de dano estético corporal.

Exame objetivo da lesão

A lesão ora objeto de análise teve como natureza Agressão física ocorrido em 15/05/2017. O prejuízo estético consiste em lesão cicatricial consolidada na região: Rosto no lado superior.

Trata-se de cicatriz Hipertrófica de tamanho/frequência Única: Retilínea de orientação Horizontal de pigmentação Normocrômica medindo centímetro(s) na horizontal, centímetros na vertical e 2 milímetros de Profundidade. De contornos Regulares. Perceptivel em 1 centímetros de distância nas relações íntimas. Perceptivel em 2 metros nas relações sociais. A lesão tem repercução na vida diária familiar conforme fotos anteriores e atuais..

Conclui-se que classificada segundo os parâmetros de dano estético AIPE - Análise do Impacto do Prejuízo Estético (escala de seis níveis e pontuação entre 0 e 50 pontos), como sendo dano estético relativo à área 1, a lesão é caracterizada por 1 pontos e grau de prejuízo estético: Leve.

Escore final: 1;
Grau de prejuízo: Leve.

, 16/05/2017

____

Me. Geraldo Elias Miranda
